# Dairy bull sperm subpopulation behaviour in frozen-thawed semen across breed, temperature, and thawing time

**DOI:** 10.17221/56/2025-VETMED

**Published:** 2026-01-26

**Authors:** Francisco Sevilla, Ignacio Araya-Zuniga, Juan Manuel Solis, Carine Corcini, Patricia Cervantes-Acosta, Antonio Hernandez-Beltran, Rafael Molina-Montero, Derling Pichardo-Matamoros, Anthony Valverde

**Affiliations:** ^1^Doctorado en Ciencias Naturales para el Desarrollo (DOCINADE), Instituto Tecnológico de Costa Rica, Universidad Nacional, Universidad Estatal a Distancia, Alajuela, Costa Rica; ^2^Laboratory of Animal Reproduction, School of Agronomy, Costa Rica Institute of Technology, Alajuela, Costa Rica; ^3^Veterinary School, Pelotas Federal University, Rio Grande do Sul, Brazil; ^4^School of Veterinary Medicine and Animal Husbandry, Veracruz University, Veracruz, México; ^5^Agricultural Production Program, School of Agronomy, Costa Rica Institute of Technology, Alajuela, Costa Rica; ^6^Animal Husbandry Engineering, National Agrarian University, Chontales, Nicaragua

**Keywords:** cryopreservation, dairy cattle, reproduction, spermatozoa, sperm kinematics

## Abstract

Optimising dairy cattle reproduction with assisted reproductive technologies, such as artificial insemination, requires standardised semen handling and analysis. This study evaluated the kinematic structure of the spermatozoan subpopulation in frozen-thawed Holstein and Jersey bull semen doses under different thawing protocols. We used frozen semen doses taken from four bulls of each breed. Nine semen doses were collected from each animal, for a total of 72 cryopreserved doses. Straw thawing was performed at three temperatures (35 °C, 37 °C, and 40 °C) and three times (30 s, 40 s, and 45 s). Sperm kinematic patterns were evaluated using a CASA-mot system (ISAS^®^v1). Sperm kinematic variables for each breed, temperature, and thawing time identified four subpopulations. The analysis revealed an effect of breed and semen thawing protocols (*P < *0.05) on sperm subpopulation distribution, sperm movement, and swimming patterns. Subpopulation analyses based on semen assessment are needed to further interpret the relevance and effect on fertility.

Animal reproduction is central to dairy production performance (Cheng et al. 2022). Assisted reproduction techniques, such as artificial insemination with fresh or frozen-thawed semen, improve animal genetics (Souza et al. 2016; Koch et al. 2022). Success of artificial insemination, however, depends on environmental conditions, management, nutrition, and semen quality (Barquero et al. 2021a; Koch et al. 2022). These reproductive biotechnologies require clear, validated, replicable protocols that minimise external variation in livestock systems (Mikkola et al. 2024). Studies have shown that protocols that manipulate semen during seminal sample thawing can influence sperm motility and functionality. Such variables include percentage of total and progressive sperm motility, plasma membrane integrity, and sperm kinetics (Diskin 2018; Goshme et al. 2021; Solis et al. 2024).

Semen analysis has traditionally focused on cell motility or morphology by studying the percentage of sperm with normal morphology in an ejaculate or seminal dose (Silvestre et al. 2023). With the emergence of Computer-Assisted Semen Analysis (CASA) systems that can now generate larger volumes of data with high-resolution cameras (Bompart et al. 2019; Valverde et al. 2019), sperm movement patterns can be predicted with greater accuracy and precision using multivariate statistical analysis models. This kind of analysis can better determine semen quality and predict male fertility (Martinez-Pastor et al. 2011; Hidalgo et al. 2021; Martinez-Pastor 2022). The analysis of cellular subsets within the ejaculate using multivariate data analysis has, furthermore, allowed us to identify sperm subpopulations within an ejaculate (Valverde et al. 2016; Martinez-Pastor 2022). These subpopulations exhibit different movement patterns that reorganise themselves based on intrinsic and extrinsic factors (Murphy et al. 2018) indicative of different sperm functions (Martinez et al. 2013; Santolaria et al. 2015; Ibanescu et al. 2018; Azevedo et al. 2024).

Sperm subpopulations of various ruminants have been classified based on motility and kinematic characteristics (Perez-Marin et al. 2021; Kanno et al. 2023), morphometry (Maroto-Morales et al. 2012; Valverde et al. 2016; Viquez et al. 2023), as well as other external factors (Garcia-Herreros and Leal 2014) such as sexual status (Araya-Zuniga et al. 2024), ejaculate fractions (Perez-Marin et al. 2021), species (Gacem et al. 2021), season (Ibanescu et al. 2018) and age (Marti et al. 2011; Menon et al. 2011). With subpopulation classification, we can now analyse sperm distribution within the ejaculate and identify animals that harbour sperm subpopulations with distributions that favour reproductive fitness and suitability for breeding or artificial insemination (Hidalgo et al. 2022). This study evaluated the kinematic behaviour of sperm subpopulations in frozen-thawed seminal doses of Holstein and Jersey bulls with different thawing protocols.

## MATERIAL AND METHODS

### Ethical approval

The study was conducted in compliance with Costa Rica’s national regulations governing research with live animals. All procedures were performed with care to minimise stress and ensure animal welfare. Ethical approval was granted by the Committee of the Tropical Sustainable Agriculture Research and Development Center (CIDASTH-ITCR), in accordance with Section 08/2023 and Article 5.0 of DAGSC-075-2023 and CIE-206-2023. The study also complied with the ARRIVE guidelines (https://arriveguidelines.org/) for reporting standards in animal research.

### Study location, seminal doses, and thawing protocols

This study was conducted at the Costa Rica Institute of Technology’s Animal Reproduction Laboratory at the San Carlos Campus in Alajuela, Costa Rica, from April to November 2023. Semen dose quality was verified prior to cryopreservation, and only ejaculates with sperm motility above 60% and normal morphology above 75% were used. Nine cryopreserved seminal doses were collected from eight bulls (Holstein, *n* = 4; Jersey, *n* = 4), totalling 72 doses. These doses were packaged in 0.25 ml volume straws and stored in an MVE^®^ XC 20 Signature cryogenic tank (MVE Biological Solutions, Ball Ground, GA, USA). To thaw doses, we followed the methodology described in Solis et al. (2024). To thaw straws in a thermoregulated water bath (Digisytem Laboratory Instruments Inc., New Taipei City, Taiwan), three temperatures (35 °C, 37 °C, and 40 °C) and three times (30 s, 40 s and 45 s) were applied. After thawing, the contents of each straw were placed in an Eppendorf^®^ tube (Sigma-Aldrich, St. Louis, MO, USA). The samples were diluted with the commercial diluent OptiXcell^®^ (IMV, L’Aigle, France) in a 1 : 1 (vol : vol) ratio, for an average final concentration of 33.19 ± 13.46 × 10^6^/ml per straw for the Jersey breed and 21.61 ± 14.54 × 10^6^/ml per straw for the Holstein.

### Sperm kinematic assessment

To assess kinematics, a previously preheated at 37 °C sample was homogenised by gentle shaking, and then a 3 μl diluted semen subsample was extracted and placed in a Spermtrack^®^ sperm counting chamber (Proiser I+D, Paterna, Spain) with a depth of 20 μm. The counting chamber had been preheated to 37 °C on a hot plate.

A CASA-mot ISAS^®^v1 system (Integrated Semen Analysis System, Proiser I+D, Paterna, Spain) was used. The system consisted of a video camera (Proiser 782M, Proiser I+D) and a UB203 microscope (UOP/Proiser R+D) equipped with a 1× eyepiece and a 10× negative phase-contrast objective (NA 0.25). The camera recorded at 50 fps with a final resolution of 768 × 576 pixels. The sperm counting chamber containing the sample was placed on the microscope’s integrated, preheated fixed stage at 37.0 ± 0.5 °C.

Two replicates of each sample were analysed, and a total of seven microscopic fields or a minimum of 600 spermatozoa were captured for each. Kinematic analysis included rectilinear velocity (VSL, μm s^–1^), curvilinear velocity (VCL, μm s^–1^), average path velocity (VAP, μm s^–1^), and percentages of linearity (LIN = VSL/VCL × 100), straightness (STR = VSL/VAP × 100), and wobble (WOB = VAP/VCL × 100). The crossover frequency (BCF, in Hz) and lateral head displacement (ALH, in μm) were also analysed.

### Statistical analysis

Results were presented as mean ± standard error of the mean. Assumptions of normality and homoscedasticity were assessed using the Shapiro–Wilk–Levene test. Once these assumptions were verified, a repeated-means analysis of variance (ANOVA) was performed. General linear and mixed models were used to analyse sperm kinematic variables. Means were compared using the least-squares method with Bonferroni correction at *P* < 0.05. Kinematic variables (VCL, VAP, VSL, LIN, STR, WOB, ALH, and BCF) were divided into subsets by breed, temperature, and thawing time, and multivariate procedures then identified groups within each subset. Variables were standardised, followed by a principal factor analysis (PFA). PFs with an eigenvalue greater than one were extracted from the PFA using the Kaiser criterion, and the Kaiser–Meyer–Olkin (KMO) test was applied. PFs were obtained by applying the Varimax rotation method with Kaiser normalisation. A non-hierarchical K-means cluster analysis was used to identify sperm subpopulations, and ANOVA was used to evaluate statistical differences between groups obtained for all kinematic variables. A post hoc pairwise comparison of group means was performed using the Tukey–Kramer test, with *P < *0.05. All analyses were performed using IBM SPSS v23.0 for Windows (SPSS Inc., Chicago, IL, USA).

## RESULTS

PFA identified four sperm subpopulations in each breed. The Holstein subpopulation 4 (SP_4_) had the highest VCL, while SP_1_ had sperm with the highest VSL and VAP. The SPs with the most linear movements, as described by LIN, STR, and WOB, were SP_1_ and SP_3_. In the Jersey breed, the highest VCL was observed in SP_4_. The highest averages for VSL and VAP were found in SP_1_. There was variation in progressivity because the most linear and oscillating movement was exhibited by SP_3_, while the greatest straightness was exhibited by SP_1_ (Table 1).

**Table 1:** Subpopulations (mean ± SEM) according to frozen-thawed semen kinematic variables by dairy cattle breed

Variable	VCL	VSL	VAP	LIN	STR	WOB
Holstein						
S_P_1	190.46 ± 0.28^b^	113.86 ± 0.22^a^	118.69 ± 0.20^a^	59.52 ± 0.10^b^	93.97 ± 0.16^a^	62.27 ± 0.09^b^
SP_2_	97.94 ± 0.21^d^	25.73 ± 0.16^d^	44.67 ± 0.15^d^	26.11 ± 0.08^d^	57.41 ± 0.12^d^	45.95 ± 0.07^c^
SP_3_	99.13 ± 0.29^c^	67.19 ± 0.22^c^	70.81 ± 0.21^c^	66.74 ± 0.11^a^	91.23 ± 0.17^b^	71.36 ± 0.10^a^
SP_4_	213.02 ± 0.36^a^	70.08 ± 0.28^b^	95.52 ± 0.26^b^	32.50 ± 0.13^c^	71.86 ± 0.21^c^	44.90 ± 0.12^d^
						
Jersey						
SP_1_	209.52 ± 0.28^b^	131.74 ± 0.22^a^	137.69 ± 0.20^a^	62.58 ± 0.09^b^	93.40 ± 0.13^a^	65.76 ± 0.08^b^
SP_2_	100.67 ± 0.26^c^	26.30 ± 0.21^d^	47.29 ± 0.19^d^	25.88 ± 0.09^d^	55.01 ± 0.12^d^	47.19 ± 0.07^c^
SP_3_	110.91 ± 0.28^d^	79.36 ± 0.23^c^	84.06 ± 0.20^c^	70.21 ± 0.09^a^	91.28 ± 0.13^b^	75.22 ± 0.08^a^
SP_4_	250.35 ± 0.33^a^	88.90 ± 0.27^b^	116.54 ± 0.24^b^	34.88 ± 0.11^c^	74.13 ± 0.15^c^	46.72 ± 0.09^d^

^a–d^Different letters between columns indicate differences between subpopulations according to breed (*P < *0.05)

LIN = linearity index (%); SEM = standard error of the mean; SP = subpopulation; STR = straightness index (%); VAP = average path velocity (μm s^–1^); VCL = curvilinear velocity (μm s^–1^); VSL = rectilinear velocity (μm s^–1^); WOB = trajectory oscillation (%)

**Figure 1:**
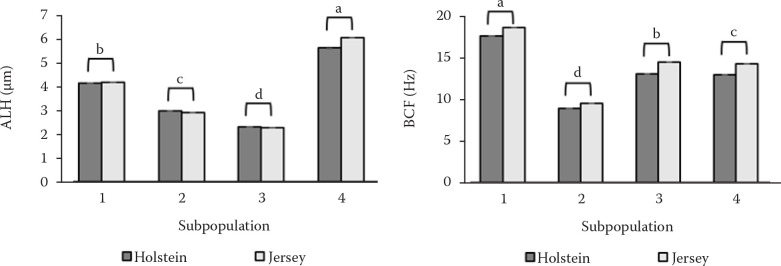
Mean oscillation values by sperm subpopulation in frozen-thawed dairy cattle semen samples ^a–d^Different letters show differences between subpopulations within each breed (*P < *0.05) ALH (μm) = amplitude of lateral head displacement; BCF (Hz) = beat-cross frequency

Significant differences in ALH were found among subpopulations by breed (*P < *0.05). Mean values in SP_4_ for both breeds were higher than in other subpopulations (*P < *0.05). SP_3_ for both Holstein and Jersey had the lowest means among subpopulations. BCF showed statistically significant differences for subpopulations within each breed (*P < *0.05). The highest BCF values were observed in SP_1_ for both, while the lowest were observed in SP_2_ (Figure 1).

Subpopulations were characterised according to semen thawing temperatures. At 35 °C, differences were observed between sperm subpopulations (*P < *0.05), with the highest values in SP_4_, followed by SP_1_, SP_3_, and SP_2_. This pattern was similar for semen thawing temperatures of 37 °C and 40 °C. Overall, SP_3_ was the subpopulation that showed the most progressive movement across all temperatures (Table 2).

**Table 2:** Subpopulations (mean ± SEM) according to kinematic variables of frozen-thawed semen and thawing temperature

Variable	VCL	VSL	VAP	LIN	STR	WOB
35 °C						
SP_1_	202.21 ± 0.37^b^	125.80 ± 0.29^a^	130.86 ± 0.27^a^	61.87 ± 0.12^b^	93.81 ± 0.18^a^	64.70 ± 0.11^b^
SP_2_	97.75 ± 0.31^d^	25.27 ± 0.24^d^	45.21 ± 0.22^d^	25.72 ± 0.10^d^	55.71 ± 0.15^d^	46.69 ± 0.09^c^
SP_3_	104.16 ± 0.38^c^	73.52 ± 0.30^c^	77.82 ± 0.27^c^	68.97 ± 0.13^a^	90.82 ± 0.19^b^	74.13 ± 0.11^a^
SP_4_	236.29 ± 0.45^a^	81.87 ± 0.35^b^	107.98 ± 0.33^b^	33.82 ± 0.15^c^	73.27 ± 0.22^c^	45.74 ± 0.13^d^
						
37 °C						
SP_1_	202.13 ± 0.34^b^	122.94 ± 0.26^a^	128.69 ± 0.24^a^	60.47 ± 0.11^b^	93.53 ± 0.16^a^	63.58 ± 0.10^b^
SP_2_	100.66 ± 0.29^d^	26.75 ± 0.22^d^	46.45 ± 0.21^d^	26.36 ± 0.10^d^	57.17 ± 0.14^d^	46.29 ± 0.08^c^
SP_3_	107.80 ± 0.36^c^	74.88 ± 0.28^c^	79.05 ± 0.26^c^	68.30 ± 0.12^a^	91.59 ± 0.17^b^	72.89 ± 0.10^a^
SP_4_	236.12 ± 0.41^a^	82.28 ± 0.31^b^	109.23 ± 0.29^b^	34.23 ± 0.14^c^	73.41 ± 0.20^c^	46.30 ± 0.12^c^

40 °C						
SP_1_	200.19 ± 0.36^b^	124.27 ± 0.29^a^	129.85 ± 0.27^a^	61.71 ± 0.13^b^	93.57 ± 0.18^a^	64.75 ± 0.11^b^
SP_2_	99.18 ± 0.30^d^	25.94 ± 0.24^d^	46.04 ± 0.22^d^	25.89 ± 0.11^d^	55.84 ± 0.15^d^	46.66 ± 0.09^c^
SP_3_	106.10 ± 0.36^c^	74.63 ± 0.28^c^	78.92 ± 0.26^c^	69.09 ± 0.12^a^	91.32 ± 0.18^b^	73.93 ± 0.11^a^
SP_4_	234.70 ± 0.46^a^	80.40 ± 0.36^b^	107.52 ± 0.34^b^	33.73 ± 0.16^c^	72.99 ± 0.23^c^	45.90 ± 0.14^d^

^a–d^Different letters between columns indicate differences between subpopulations according to semen thawing temperature (*P < *0.05)

LIN = linearity index (%); SEM = standard error of the mean; SP = subpopulation; STR = straightness index (%); VAP = average path velocity (μm s^–1^); VCL = curvilinear velocity (μm s^–1^); VSL = rectilinear velocity (μm s^–1^); WOB = trajectory oscillation (%)

**Figure 2:**
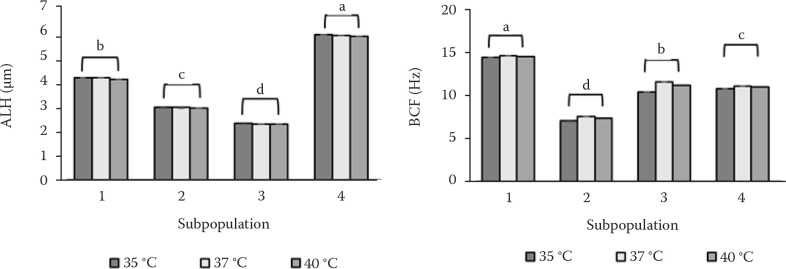
Mean sperm oscillation values obtained from frozen-thawed semen samples from dairy cattle at different thawing temperatures ^a–d^Different letters show differences between subpopulations within each thawing temperature (*P < *0.05) ALH (μm) = amplitude of lateral head displacement; BCF (Hz) = beat-cross frequency

At a thawing temperature of 35 °C, the greatest sperm undulation was observed in SP_4_ and SP_1_, with higher average ALH and BCF values, respectively. A similar pattern was observed at temperatures of 37 °C and 40 °C (Figure 2). SP_2_ and SP_3_ had the lowest average values across all analysed temperatures (*P < *0.05).

**Figure 3:**
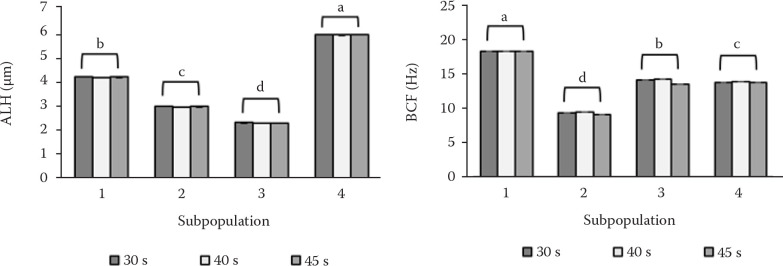
Mean sperm oscillation values obtained from frozen-thawed dairy cattle semen at different thawing times ^a–d^Different letters show differences between subpopulations within each thawing time (*P < *0.05) ALH (μm) = amplitude of lateral head displacement; BCF (Hz) = beat-cross frequency

The sperm subpopulation was analysed for thawing times (Table 3). For the 30 s thawing time, significant differences were observed between subpopulations (*P < *0.05). The SP_4_ subpopulation had the highest velocity, while the cells with the most progressive, linear movement were present in SP_3_. At the thawing times of 40 s and 45 s, very similar subpopulation behaviour patterns emerged, with the highest velocity and progressiveness observed in SP_1_ and SP_3_, respectively. There were differences between subpopulations for the three thawing temperatures (*P < *0.05) in the sperm oscillation variables, ALH and BCF; however, no differences were found (*P > *0.05) between semen thawing temperatures within subpopulations. The highest ALH values were observed in SP_4_, with values exceeding 5 μm. For BCF, the highest averages were in SP_4_ across all thawing temperatures (Figure 3).

**Table 3:** Kinematic subpopulations of frozen-thawed semen from dairy cattle according to the thawing time

Variable	VCL	VSL	VAP	LIN	STR	WOB
30 s						
SP_1_	201.18 ± 0.35^b^	123.38 ± 0.27^b^	128.96 ± 0.25^a^	60.95 ± 0.12^b^	93.57 ± 0.17^a^	64.01 ± 0.10^b^
SP_2_	100.03 ± 0.29^d^	26.19 ± 0.23^d^	46.20 ± 0.21^d^	25.94 ± 0.10^d^	56.17 ± 0.14^d^	46.42 ± 0.08^c^
SP_3_	106.66 ± 0.36^c^	74.36 ± 0.28^c^	78.59 ± 0.26^c^	68.60 ± 0.12^a^	91.33 ± 0.18^b^	73.37 ± 0.10^a^
SP_4_	236.29 ± 0.42^a^	81.70 ± 0.32^a^	108.33 ± 0.30^b^	33.97 ± 0.14^c^	73.37 ± 0.20^c^	45.93 ± 0.12^d^
						
40 s						
SP_1_	201.64 ± 0.36^b^	124.84 ± 0.29^a^	130.24 ± 0.26^a^	61.60 ± 0.12^b^	93.37 ± 0.17^a^	64.56 ± 0.11^b^
SP_2_	99.04 ± 0.32^d^	26.18 ± 0.25^d^	45.74 ± 0.23^d^	26.22 ± 0.11^d^	56.88 ± 0.15^d^	46.39 ± 0.09^c^
SP_3_	107.71 ± 0.38^c^	75.92 ± 0.30^c^	80.31 ± 0.28^c^	69.06 ± 0.13^a^	91.38 ± 0.18^b^	73.94 ± 0.11^a^
SP_4_	235.58 ± 0.46^a^	82.47 ± 0.36^b^	108.73 ± 0.33^b^	34.37 ± 0.16^c^	73.85 ± 0.20^c^	46.21 ± 0.13^c^
						
45 s						
SP_1_	201.88 ± 0.36^b^	124.66 ± 0.28^a^	130.11 ± 0.26^a^	61.39 ± 0.12^b^	93.61 ± 0.18^a^	64.35 ± 0.11^b^
SP_2_	98.57 ± 0.29^d^	25.66 ± 0.23^d^	45.77 ± 0.21^d^	25.86 ± 0.10^d^	55.83 ± 0.15^d^	46.80 ± 0.09^c^
SP_3_	104.05 ± 0.36^c^	73.00 ± 0.28^c^	77.15 ± 0.26^c^	68.73 ± 0.12^a^	91.07 ± 0.18^b^	73.64 ± 0.11^a^
SP_4_	235.30 ± 0.44^a^	80.50 ± 0.34^b^	107.94 ± 0.32^b^	33.54 ± 0.15^c^	72.53 ± 0.22^c^	45.91 ± 0.13^c^

^a–d^Different letters between columns indicate differences between subpopulations according to the thawing time of seminal doses (*P < *0.05)

LIN = linearity index (%); SEM = standard error of the mean; SP = subpopulation; STR = straightness index (%); VAP = average path velocity (μm s^–1^); VCL = curvilinear velocity (μm s^–1^); VSL = rectilinear velocity (μm s^–1^); WOB = trajectory oscillation (%)

## DISCUSSION

Seminal doses used in assisted reproduction processes exhibit significant variations in movement patterns and kinematics, as evidenced by groupings within each dose (Muino et al. 2008; Muino et al. 2009; Yaniz et al. 2018; Ibanescu et al. 2020). These variations may be influenced by individual variables (e.g., breed) and ejaculate handling (temperature and thawing time) (Valverde et al. 2016). Variable sperm behaviour in *Bos taurus* bull ejaculates across breeds has already been reported (Menon et al. 2011; Ntemka et al. 2016). This variability contributes to its adaptability to the environmental conditions in which individuals develop. The protocols used to thaw semen can provoke external variation in kinematic cell patterns (Alvarez-Rodriguez et al. 2024; Solis et al. 2024). The results of this work indicate that different semen temperatures and thawing times affect swimming patterns; furthermore, this may affect the probability of sperm fertilising once doses are used (Koch et al. 2022).

Sperm motility is a principal fertility indicator, since the sperm, once it enters the female reproductive tract, must traverse a long gauntlet of physical and chemical obstacles to reach the ampulla (Ntemka et al. 2016). Previous studies show that Holstein and Jersey breeds exhibit differences in sperm motility and kinematic patterns (Solis et al. 2024); however, subpopulation characterisation was not done. This underscores the utility of subpopulation dynamics analysis for interpreting the biological implications of ejaculate heterogeneity and its potential impact on fertility (Valverde et al. 2019; Azevedo et al. 2024; Martinez-Pastor 2022). These analyses elucidate male fertility by understanding sperm behaviour within the ejaculate and its potential functionality (Keyer et al. 2022). This knowledge provides a more objective criterion for selecting animals to participate in genetic improvement programs at artificial insemination centres (Ferraz et al. 2014).

Previous cluster analysis and identification of sperm subpopulations within ejaculate fractions (Valverde et al. 2016; Perez-Marin et al. 2021) have demonstrated ejaculate variability and the need to select among those fractions for the greatest preservation value. This discovery has recently led to numerous studies linking fertility to sperm subpopulation dynamics (Hidalgo et al. 2021; Hidalgo et al. 2022; Alm-Kristiansen 2023). It has further allowed us to associate swimming patterns, speed, and linearity with higher fertility rates (Ahmed et al. 2016; Yaniz et al. 2016; Pena et al. 2018). Other studies have shown that low-fertility males suffer from ejaculate subpopulations dominated by slow, non-linear movements (Ramon et al. 2013).

Some authors hypothesise that different subpopulations perform different functions within the ejaculate (Ibanescu et al. 2020). This functionality and group distribution vary depending on ejaculate-handling conditions and breed (Garcia-Alvarez et al. 2014; Alm-Kristiansen 2023). Overall, this study shows that kinematic differences are higher in two of the four identified subpopulations. These variations may be due to the individual nature of the animal and its ejaculate per se (Fernandez-Lopez et al. 2022), as well as different functional subpopulations within the ejaculate and sperm maturity (Alm-Kristiansen 2023). Despite these advancements, it is still not possible to precisely determine the effects of these functional subpopulations on fertility (Barquero et al. 2021b). Our results also show that sperm distribution and kinematic patterns in subpopulations were more progressive and linear, depending on the thawing time and temperature protocol used, an observation not previously reported. This new evidence calls for additional study of these variables and their potential impact on sperm function, especially under tropical conditions.

In conclusion, the aim of this study was to investigate the effect of animal breed and semen thawing protocols on sperm subpopulation dynamics. Based on the data from this study, we observed that within the practical ranges tested (35–40 °C and 30–45 s), changing thawing temperature or thawing time does not eliminate or fundamentally alter the key progressive subpopulations (SP_1_ and SP_3_). This suggests that the structure of post-thaw sperm motility and kinematics is robust to small protocol variations and that the functionally relevant progressive fraction is preserved. Subpopulation analysis is needed to improve the interpretation of its biological relevance and effect on fertility.
